# Grapefruit Juice Flavanones Modulate the Expression of Genes Regulating Inflammation, Cell Interactions and Vascular Function in Peripheral Blood Mononuclear Cells of Postmenopausal Women

**DOI:** 10.3389/fnut.2022.907595

**Published:** 2022-05-26

**Authors:** Irena Krga, Karla Fabiola Corral-Jara, Nicolas Barber-Chamoux, Claude Dubray, Christine Morand, Dragan Milenkovic

**Affiliations:** ^1^Centre of Research Excellence in Nutrition and Metabolism, Institute for Medical Research, National Institute of Republic of Serbia, University of Belgrade, Belgrade, Serbia; ^2^Université Clermont Auvergne, INRAE, UNH, Clermont-Ferrand, France; ^3^Institut National de la Santé et de la Recherche Médicale (INSERM), CIC 501, UMR 766, Clermont-Ferrand, France; ^4^Department of Nutrition, College of Agricultural and Environmental Sciences, University of California, Davis, Davis, CA, United States

**Keywords:** naringin, flavanone, genomics, bioinformatics, vascular function

## Abstract

Grapefruit is a rich source of flavanones, phytochemicals suggested excreting vasculoprotective effects. We previously showed that flavanones in grapefruit juice (GFJ) reduced postmenopausal women’s pulse-wave velocity (PWV), a measure of arterial stiffness. However, mechanisms of flavanone action in humans are largely unknown. This study aimed to decipher molecular mechanisms of flavanones by multi-omics analysis in PBMCs of volunteers consuming GFJ and flavanone-free control drink for 6 months. Modulated genes and microRNAs (miRNAs) were identified using microarrays. Bioinformatics analyses assessed their functions, interactions and correlations with previously observed changes in PWV. GFJ modified gene and miRNA expressions. Integrated analysis of modulated genes and miRNA-target genes suggests regulation of inflammation, immune response, cell interaction and mobility. Bioinformatics identified putative mediators of the observed nutrigenomic effect (STAT3, NF-κB) and molecular docking demonstrated potential binding of flavanone metabolites to transcription factors and cell-signaling proteins. We also observed 34 significant correlations between changes in gene expression and PWV. Moreover, global gene expression was negatively correlated with gene expression profiles in arterial stiffness and hypertension. This study revealed molecular mechanisms underlying vasculoprotective effects of flavanones, including interactions with transcription factors and gene and miRNA expression changes that inversely correlate with gene expression profiles associated with cardiovascular risk factors.

**Clinical Trial Registration:**

[ClinicalTrials.gov], identifier [NCT01272167].

## Introduction

Grapefruit is a citrus fruit regularly consumed worldwide as fresh fruit or juice due to its taste, high nutritional value and suggested health-promoting properties. Consumption of grapefruit has been associated with a reduced risk of coronary artery disease (CAD)-related mortality and lower plasma levels of inflammatory biomarkers in postmenopausal women (1, 2). Moreover, these fruits have been linked with bodyweight management, improvements in lipid profiles, endothelial function, insulin resistance, and anti-inflammatory, antioxidant, anti-bacterial and anti-carcinogenic activities (3–5). These benefits may be ascribed to its content in micronutrients, like vitamin C, folic acid, potassium and calcium, but also to its richness in polyphenols.

Polyphenols in grapefruit are predominantly flavanones (6). Dietary intake of flavanones has been inversely associated with the risk of ischemic stroke and cerebrovascular diseases (7–9). Moreover, these compounds also displayed hypolipidemic, anti-inflammatory, antiatherogenic, vasculoprotective, anti-carcinogenic and neuroprotective properties in numerous animal and *in vitro* studies and a few clinical trials (10–12). Flavanones in citrus fruits are predominantly present as glycosides, which must be hydrolyzed into aglycones by gut microbiota before their absorption. Absorbed aglycones are then conjugated by intestinal and hepatic phase II enzymes to release glucuronide and sulfate derivatives in plasma (10, 11). Unabsorbed aglycones are further processed by gut microbiota into highly absorbable low molecular weight phenolic compounds (10). Therefore, the flavanones present in circulation are not native forms but various metabolites that mediate the beneficial effects of dietary flavanones.

The most notable flavanones in grapefruit are glycosides of naringenin, namely naringin and, to a lesser extent, narirutin (6). Several lines of clinical and preclinical evidence support the role of grapefruit flavanones in cardiovascular protection. For example, naringin was reported to improve blood cholesterol levels and induce antioxidant enzymes in hypercholesterolemic subjects (13). Moreover, we have shown previously that the consumption of flavanones in grapefruit juice for 6 months protects from arterial stiffening in postmenopausal women, as evidenced by reduced pulse wave velocity (PWV) (14). Also, a reduced atherosclerotic plaque formation, plasma cholesterol and biomarkers of endothelial dysfunction have been reported in a murine model of atherosclerosis supplemented with a diet enriched in naringin (15).

Mechanistic studies revealed that naringin’s beneficial effects are achieved through interactions with cell signaling pathways and modulation of gene and protein expressions (16, 17). Transcriptomic analysis of the aorta of mice fed naringin suggested that the antiatherogenic effect of this flavanone is due to modulations of the expression of genes regulating inflammation, adhesion and transmigration of immune cells to ultimately preserve vascular integrity (15). Similar modulations were observed in endothelial cells exposed to naringin and its plasma phase II metabolites (i.e., naringenin-4′-glucuronide and naringenin-7-glucuronide) before tumor necrosis alpha (TNFα)-induced inflammation (18). These gene expression changes were accompanied by reduced adhesion of monocytes to TNFα-activated endothelial cells, which was more pronounced when both endothelial and immune cells were exposed to plasma naringin metabolites. This finding suggests the potency of these compounds to maintain vascular integrity by acting on circulating immune cells. Moreover, microRNAs (miRNA) i.e., small non-coding RNAs that regulate gene expression at the post-transcriptional level, have emerged in recent years as a possible target of polyphenols to exert their cardioprotective properties (19). Previously, a nutritionally relevant dose of naringin was shown to modulate miRNA expression levels in atherosclerotic animals, suggesting that the biological effects of this grapefruit flavanone can involve miRNA regulation (20).

Beyond animal and *in vitro* studies reporting cardiovascular disease (CVD)-related nutrigenomic effect of flavanones, the nutrigenomic data in humans are scarce. Therefore, this study aimed to analyze the effect of the chronic consumption of flavanones in grapefruit juice (GFJ) on the global gene and miRNA expression profiles in peripheral blood mononuclear cells (PBMCs) of healthy postmenopausal women to better understand the molecular mechanisms mediating the cardiovasculoprotective effects of flavanones.

## Materials and Methods

### Study Design

The present work examines the nutrigenomic effects of flavanones in GFJ in human PBMCs. This study builds on a randomized, controlled, 6-month dietary intervention trial, registered at ClinicalTrials.gov under identifier NCT01272167 that assessed the contribution of flavanones in the chronic effects of GFJ on vascular protection in healthy postmenopausal women, with results published elsewhere (14). This clinical trial was a cross-over with two arms, a flavanone-rich GFJ and a control drink without flavanones, separated by a 2-month washout period. The trial was conducted at the Clinical Research Unit of the University Hospital of Clermont-Ferrand (France) and was approved by the local Human Ethics Committee (CPP Sud-Est VI, France).

### Subjects

Non-smoking, healthy Caucasian women, 50–65 years old and 3 to 10 years after menopause, were recruited from the Clermont-Ferrand region in France (14). The subjects were eligible for inclusion if presented a normal to overweight body mass index (19–29 kg/m2), waist circumference over 88 cm, normal blood pressure levels, normal blood analyses at screening, and normal baseline electrocardiogram examination with a corrected Q-T interval of less than 420 ms. Exclusion criteria included a medical history of metabolic diseases, cancer, or major gastrointestinal surgery, smoking, treatment with blood lipid-lowering drugs, anti-hypertensive drugs, CYP3A4-metabolized drugs or other drugs that may interfere with GFJ consumption (21), hormone replacement therapy within 3 previous months, allergy or intolerance to citrus-containing foods, use of dietary supplements (e.g., antioxidants and phytoestrogens), special dietary habits (e.g., vegetarianism and veganism) or physical activity level greater than 5 h per week. Subjects were also excluded if their habitual consumption of one or more flavonoid-rich drinks, such as coffee, tea, cocoa, wine, or fruit juices, surpassed 250 ml/day, as estimated at screening from the diet history interview. Written informed consent was obtained from each of the study participants.

### Interventional Treatments

Study participants (*N* = 48) were randomly assigned to consume 340 ml daily dose of GFJ, providing approximately 210 mg of naringenin glycosides corresponding to 105 mg naringenin (aglycone), or the same volume of a matched, isoenergetic control drink without flavanones. Concentrate of blond GFJ was provided by the Florida Department of Citrus (Lake Alfred, FL, United States) and the control drink powder mix was from PYC-DENA Laboratory (Eupen, Belgium). Both beverages were prepared and packed by Ocean Spray (Lakeville-Middleboro, MA, United States). A detailed composition of the study beverages is presented elsewhere (14). Study treatments were provided in bottles blinded at the time of packaging and participants were asked to keep them at low temperature in the dark. During the study, subjects were instructed to maintain their dietary habits but minimize their consumption of citrus foods and flavonoid-rich beverages (coffee, tea, cocoa, wine and fruit juices). Compliance with treatment was evaluated by counting bottles collected at the end of each intervention period and comparing vitamin C plasma levels at the beginning and the end.

### Blood Collection and Peripheral Blood Mononuclear Cell Isolation

For this nutrigenomic study, blood samples were collected from 12 volunteers randomly selected at the end of each 6-month dietary intervention. Blood was drawn in the morning after an overnight fast using heparin vacutainer tubes (Becton Dickinson, Franklin Lakes, NJ, United States) and immediately centrifuged for 20 min at 1,500*g* at room temperature. The cell layer was collected and washed twice with sterile PBS, with centrifugation for 10 min at 300 *g* between each washing step. The obtained pellet of PBMCs was immediately frozen and kept at −80°C until used.

### Total RNA Extraction

The PBMCs were lysed using a lysing buffer solution from the RNeasy Micro Kit (Qiagen, Hilden, Germany). Total RNA extraction was performed using the RNeasy Micro Kit as recommended by the manufacturer. RNA quality was assessed by 1% agarose gel electrophoresis and quantity determined by measuring absorbance at 260 and 280 nm on NanoDrop ND-1000 spectrophotometer (Thermo Scientific, Wilmington, DE, United States). The total RNA was stored at −80°C until analyzed.

### Gene Expression Analysis

Fifty ng of total RNA extracted were amplified and fluorescently labeled to produce Cy5 or Cy3 cRNA using the Low Input Quick Amp Labeling two color Kit (Agilent, Santa Clara, CA, United States) in the presence of spike-in two color control, as recommended by the manufacturer. Following the purification, 825 ng of labeled cRNA were hybridized onto G4845A Human GE 4 × 44K v2 microarray (Agilent, Santa Clara, CA, United States) containing 27958 Entrez Gene RNAs sequences. Microarrays were scanned with Agilent G2505 scanner (Agilent, Santa Clara, CA, United States) and data extracted by Feature Extraction software (Agilent, Santa Clara, CA, United States) version 11.0 using linear and Lowess normalization. Statistical analyses were performed using the free R 2.1 software (http://www.r-project.org). Data were analyzed by Student’s *t*-test to detect differentially expressed genes, and the probability values were adjusted by Benjamini-Hochberg correction for multiple testing at 0.1 to eliminate false positives.

### miRNA Expression Analysis

miRNA was labeled using the miRNA labeling and hybridization kit from Agilent technologies (Agilent, Santa Clara, CA, United States) as recommended by the manufacturer. Briefly, 100 ng of each total RNA sample were treated with calf intestinal phosphatase for 30 min at 37°C before denaturation with pure dimethyl sulfoxide at 100°C for 5 min and rapid transfer in ice water bath to prevent RNA reannealing. RNA samples were labeled with pCp-Cy3 using T4 RNA ligase by incubation for 2 at 16°C h, purified with microBioSpin columns, and hybridized to human 8 × 15K miRNA arrays (Agilent, Santa Clara, CA, United States) for 20 h at 55°C. Microarrays were washed in GE Wash Buffer 1 and 2 (Agilent, Santa Clara, CA, United States), scanned with Agilent Microarray Scanner (Agilent, Santa Clara, CA, United States) and data were extracted by Feature Extraction Software (Agilent, Santa Clara, CA, United States). Genespring GX10 software (Agilent, Santa Clara, CA, United States) was used to quantify the signal and background intensity for each feature, substantially normalize the data by the 75th percentile method and perform paired *t*-tests. The statistical significance was the corrected ratios of hybridization signal intensity between samples from subjects consuming GFJ and a control drink.

### Biological Interpretation of Differentially Expressed Genes

The bioinformatic analyses of nutrigenomic data have been carried out using different tools. Default parameters were used unless specified otherwise. Ensembl database^1^ was used to identify the official symbols and names of significantly modulated genes. Gene expression profiles in subjects drinking GFJ and control drink were compared by partial least squares-discriminant analysis (PLS-DA) using the MetaboAnalyst web-based software.^2^ ShinyGO online tool,^3^ version 0.61 was used for Gene Ontology Enrichment Analysis and visualization to identify biological processes related to the identified differentially expressed genes. Process network analysis was performed with the text mining algorithm of MetaCore software suite (Clarivate Analytics, Philadelphia, PA, United States).^4^ GeneTrail2, version 1.6^5^ (22) that allows accessing BioCarta, Kyoto Encyclopedia of Genes and Genomes (KEGG) and NCI Pathways databases was used for the identification of enriched pathways using the over-representation analysis and Benjamini-Yekutieli false discovery rate (FDR) correction. STRING database, version 11.0^6^ (23) was used to analyze protein-protein interactions (PPI) between proteins encoded by differentially expressed genes, including their neighboring proteins. Active interaction sources included text-mining, experiments, databases, and co-expression and network edges displayed confidence. The minimum required interaction score was set to high confidence (0.700), displaying query proteins only in the 1st shell and no more than 5 interactions in the 2nd shell. PPI enrichment *p*-value was computed by STRING network analysis default model that is based on a random background model that preserves the degree distribution of the proteins in a given list (Random Graph with Given Degree Sequence). Transcription factors that could potentially regulate the identified differentially expressed genes were obtained using the EnrichR platform^7^ that accesses the TRRUST database on transcription factor-target interactions (2019) with *p*-values generated by Fisher’s exact test. Potential binding interactions between top transcription factors and naringin metabolites, were examined by molecular docking using the Mcule docking analysis tool.^8^ Protein 3D structures were obtained from Protein Data Bank (www.rcsb.org) and chemical structures of naringin metabolites from PubChem database.^9^ Experimentally validated and computationally predicted miRNA-target interactions downloaded from miRTarBase website Release 7.0 and TargetScan website Release 7.2, were assessed trough MIENTURNET online tool^10^ (24) to obtain the list of target genes of differentially expressed miRNAs. Interactions between functional groups of genes, interactions between the identified transcription factors and genes they regulate, and miRNA and their target genes were analyzed with Cytoscape software (version 3.7.2)^11^ by the ClueGO (25).

### Correlation Analysis

Pearson’s correlation analysis was conducted with 1930 filtered probe sets as a basis with software R 3.1.0,^12^ and the Hmisc package. Correlation coefficients ρ (rho) range from −1 to +1. Values larger than zero display a positive relationship between gene expression and parameter values, whereas values smaller than zero indicate a negative relationship. Multiple test correction according to Benjamini-Hochberg was applied. For further interpretation of data, a *p*-value <0.05 was considered significant for correlations between transcript levels and PWV. The correlations were represented in a scatterplot matrix.

## Results

### Grapefruit Juice Consumption Modulates the Expression of Genes in Peripheral Blood Mononuclear Cells in Human

**FIGURE 1:**
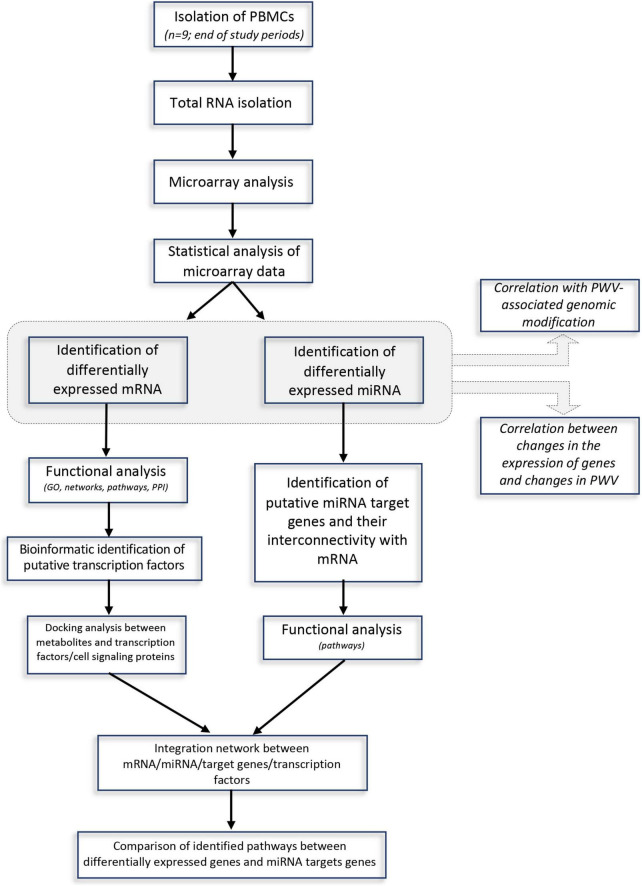
A flow-chart describing steps implemented in the genomic and bioinformatic analysis.

**FIGURE 2:**
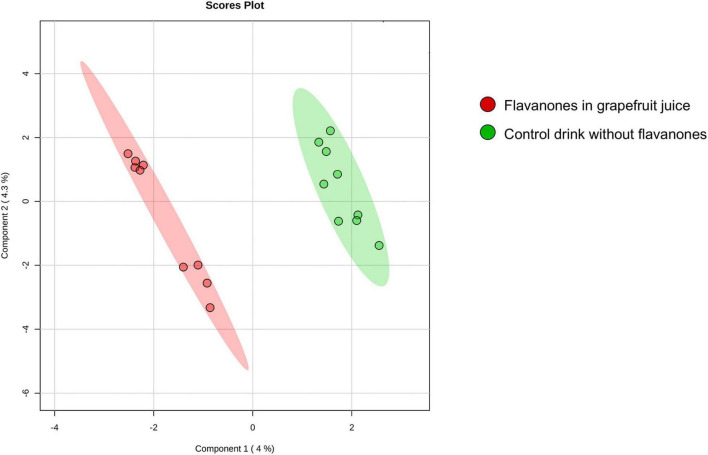
A 2D principal component analysis plot of the genomic profiles of PBMCs from volunteers after consumption of flavanones in GFJ and same volunteers after control drink consumption.

The approach used in this study, including RNA extraction, microarray analysis and different bioinformatic analyses, is presented in Figure 1. After checking for RNA and hybridization quality, a total of 9 samples per group were included in the gene expression analyses. A comparison of global gene expression profiles in the PBMCs of subjects drinking GFJ and control drink by PLS-DA demonstrated the separation among these experimental groups (Figure 2). This analysis revealed a differential nutrigenomic modulation after the chronic GFJ consumption compared to the control drink. Statistical analysis of the obtained gene expression data showed that GFJ intervention induced significant changes in PBMC gene expression, with 1,930 probes identified as differentially expressed between the two experimental groups corresponding to 1,636 different genes. After applying a cut-off (1.05 ≤fold change ≥−1.05), there were 1,401 differentially expressed genes, of which 671 genes were identified as down-regulated and 730 as upregulated by chronic GFJ consumption. The fold-change values varied from −1.35 to 1.43 (Supplementary Table 1).

### Functional Ontology, Network, and Pathway Analyses of Differentially Expressed Genes

**FIGURE 3:**
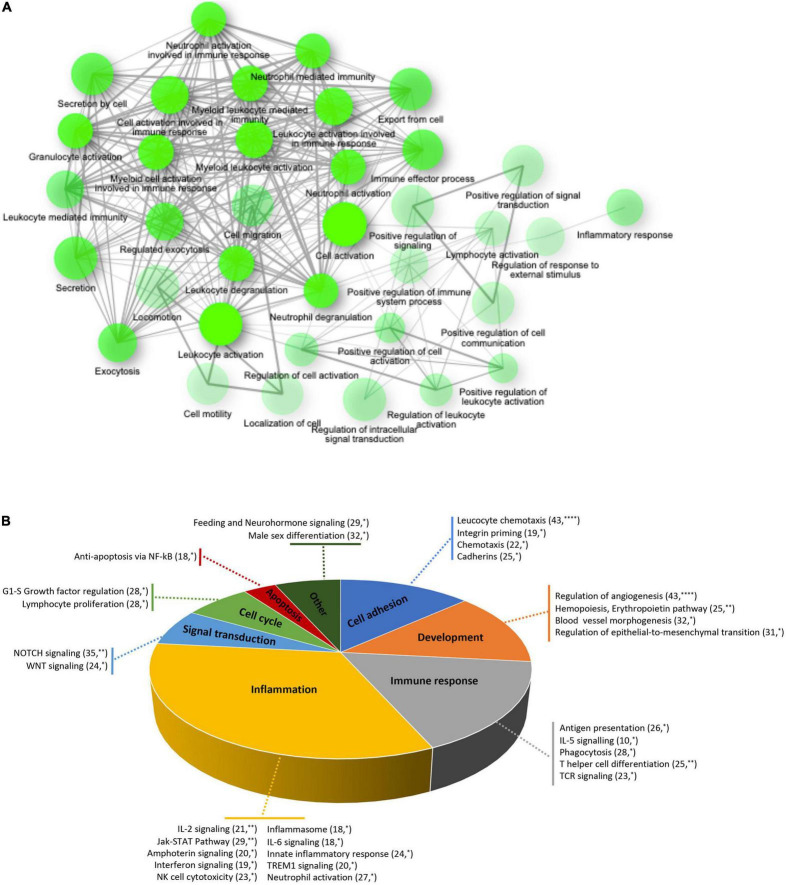
Functional ontology and network analyses of differentially expressed genes. **(A)** Network of over-represented gene ontologies of identified differentially expressed genes identified using ShinyGO tool. Two nodes are connected if they share 20% or more genes. Darker nodes are more significantly enriched gene sets. Bigger nodes represent larger gene sets. Thicker edges represent more overlapped genes. **(B)** Pie chart of significantly over-represented functional gene networks identified using text-mining approach from Metacore. Pie chart represent functional groups of the identified networks, with section proportional to the number of networks in each group. Gene networks of each functional group are represented together with the number of genes in each network. *p < 0.05, ^**^p < 0.01, and ^****^p < 0.0001.

To elucidate the biological functions of the differentially expressed genes, a Gene Ontology enrichment analysis was performed. It showed that chronic GFJ consumption modulated the expression of genes involved in different biological processes, including immune responses, inflammation, chemotaxis and cell motility (Figure 3A).

A process network analysis based on a text mining approach was further performed using Metacore to deepen the understanding of functions related to significantly modulated genes. Like for gene ontology analysis, these results suggested that regular consumption of GFJ modulated the expression of genes implicated in inflammation, immune response, cell adhesion, development, signal transduction, cell cycle and apoptosis (Figure 3B). Inflammation-related cluster included IL-2, IL-6 signaling, innate inflammatory response, inflammasome, neutrophil activation, and interferon signaling networks. The immune response included antigen presentation, IL-5 signaling, T helper cell differentiation, phagocytosis and TCR signaling networks. Within the cell adhesion processes were networks regulating leukocyte chemotaxis, integrin priming, and cadherins, and the development process included networks implicated in angiogenesis, hemopoiesis, and blood vessel morphogenesis. The signal transduction network cluster encompassed networks such as NOTCH and WNT signaling. Additionally, apoptosis included anti-apoptosis mediated by external signals *via* NF-κB, and cell growth and proliferation cluster encompassed G1-S growth factor regulation and lymphocyte proliferation networks.

**FIGURE 4:**
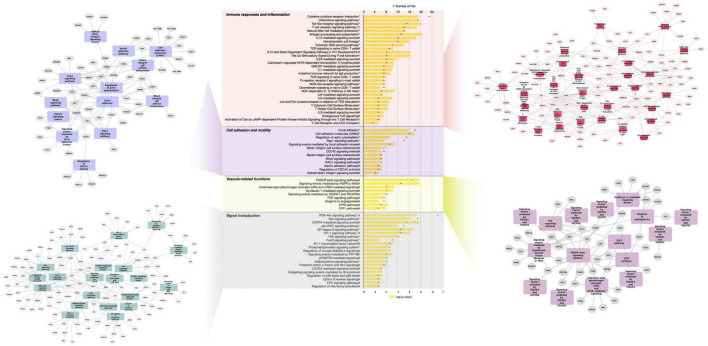
Functional pathway analysis of differentially expressed genes presented as a histogram of significantly over-represented pathways and networks of genes present in pathways of functional groups. Yellow bars depict *p*-values of the identified significant pathways, and orange dots the number of genes in each pathway. Histogram data obtained using BioCarta (†), Kyoto Encyclopedia of Genes and Genomes (*) and NCI Pathways (#) databases through GeneTrail2. Networks of genes present in pathways of functional groups obtained using Cytoscape.

Further analysis of differentially expressed genes by GeneTrail2 revealed cellular pathways associated with GFJ-modulated genes. Once again, the overrepresented pathways included those implicated in immune system responses, inflammation, cell adhesion and motility, vasculo-related functions, signal transduction, and metabolism (Figure 4 and Supplementary Figure 1). Among the immune response and inflammation-associated pathways, the highest number of genes modulated by GFJ were found in Cytokine-cytokine receptor interaction pathway, Chemokine signaling pathway, Toll-like receptor signaling pathway, T cell receptor signaling pathway and Antigen processing and presentation. Top pathways associated with cell adhesion and motility included Focal adhesion, Cell adhesion molecules (CAMs), Regulation of actin cytoskeleton and Rap1 signaling pathway. Among vasculo-related pathways, the overrepresented pathways included PDGFR-beta signaling pathway, signaling events mediated by Hepatocyte Growth Factor Receptor (c-Met), Syndecan-1-mediated signaling events, Signaling events mediated by VEGFR1 and, and Urokinase-type plasminogen activator (uPA) and uPAR-mediated signaling. The most notable pathways among signal transduction were PI3K-Akt signaling pathway, Ras signaling pathway, CXCR4-mediated signaling events and NF-kappa B signaling pathway. Overrepresented pathways also included those associated with cellular metabolism, including Glycerophospholipid metabolism, Carbon metabolism and Glycolysis/gluconeogenesis, as well as other cellular functions like Direct p53 effectors, Endocytosis and Lysosome.

Overall, these analyses suggested that GFJ flavanones modulate the expression of genes involved in regulation of immune response, inflammation, cell motility and signal transduction.

### Protein-Protein Interactions

A network of PPI was built from the obtained differentially expressed genes with the aim to identify nodes (proteins) with the highest number of functional connections, whose modulation may affect protein-protein interactome and associated cellular functions. The PPI network from differentially expressed genes, presented in Supplementary Figure 2A, included 1,286 nodes and 2,952 edges (predicted functional associations), with a PPI enrichment value <1.0e–16. Further inspection of this network showed that C3AR1, CXCL1, CXCL10, and BTRC displayed the highest interactions within the obtained network (Supplementary Figure 2B). The analysis of top identified nodes (≥10 interactions) by GeneTrail2 suggested their involvement in several pathways, including chemokine signaling, T cell receptor signaling, toll-like receptor signaling and TNF signaling.

### Transcription Factors Potentially Involved in the Nutrigenomic Effect of Grapefruit Juice Flavanones

The next step of bioinformatic analyses was to use differentially expressed genes to identify transcription factors potentially mediating the observed nutrigenomic effect of flavanone-rich GFJ consumption. The most significant transcriptional regulators identified included STAT3, STAT1, TRP53, ETV4, RUNX1, and MEF2D (Supplementary Table 2). The interactions between the identified transcription factors and differentially expressed genes are presented in Supplementary Figure 3. This analysis showed interconnections between transcription factors and differentially expressed genes, forming one network of interactions. It also revealed a few major nodes of interactions, that is, transcription factors presenting a high number of interactions with the identified differentially expressed genes. Among these nodes are NF-κB, SP1 and STAT3. This observation suggests that these transcription factors could be involved in the nutrigenomic modifications induced by GFJ consumption.

### Docking Analyses Between Naringin Metabolites and Potential Cell Signaling Proteins Involved in the Nutrigenomic Effect

**FIGURE 5:**
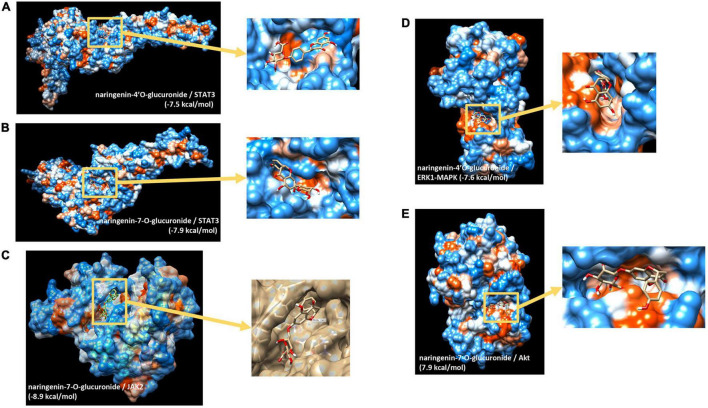
*In silico* docking analysis of interactions between naringin metabolites and potential transcription factors and cell signaling proteins. **(A)** Naringenin 4’-O-glucuronide to STAT3; **(B)** naringenin-7-O-glucuronide to STAT3; **(C)** naringenin-7-O-glucuronide to Jak2; **(D)** naringenin 4’-O-glucuronide to ERK1(MAPK3); **(E)** naringenin-7-O-glucuronide to Akt.

We then aimed to identify potential capacity of major naringin metabolites present in the circulation to interact and bind to several transcription factors identified and cell signaling proteins involved in the activity of these transcription factors. Using *in silico* docking modeling, we observed that naringenin-4’O-glucuronide present potential binding capacity of −7.5 kcal/mol to STAT3 (Figure 5A) as well as naringenin-7-O-glucuronide (−7.9 kcal/mol) (Figure 5B). These observations suggest that 2 major naringin metabolites present a potential capacity of interacting with STAT3 transcription factors. Moreover, we also analyzed possible interactions between naringin metabolites and cell signaling proteins involved in regulating the activity of transcription factors identified using bioinformatic analysis. Using this approach, a potential high probability of interaction between naringenin-7-O-glucuronide and JAK2 signaling protein (−8.9 kcal/mol) (Figure 5C) that regulates, among others, the activity of STAT3 was detected. We also observed that naringenin-4’O-glucuronide presents a potential probability of interacting with ERK1-MAPK signaling protein (−7.6 kcal/mol) (Figure 5D), a protein that regulates activation of STAT3 but also STAT1 and NF-κB. Furthermore, naringenin-7-O-glucuronide could also interact with Akt signaling protein, involved in the regulation of NF-κB, with a free minimal energy of 7.9 kcal/mol (Figure 5E). However, these analyses do not allow us to identify if potential interactions result in activation or inhibition of activities of target proteins. These *in silico* analyses suggest that flavanone metabolites may bind to transcription factors and cell signaling proteins, with as a potential consequence, modifications of their kinase activity and activation of downstream cell signaling proteins and transcription factors.

### Grapefruit Juice Flavanones Modulate the Expression of miRNA in Peripheral Blood Mononuclear Cells

**FIGURE 6:**
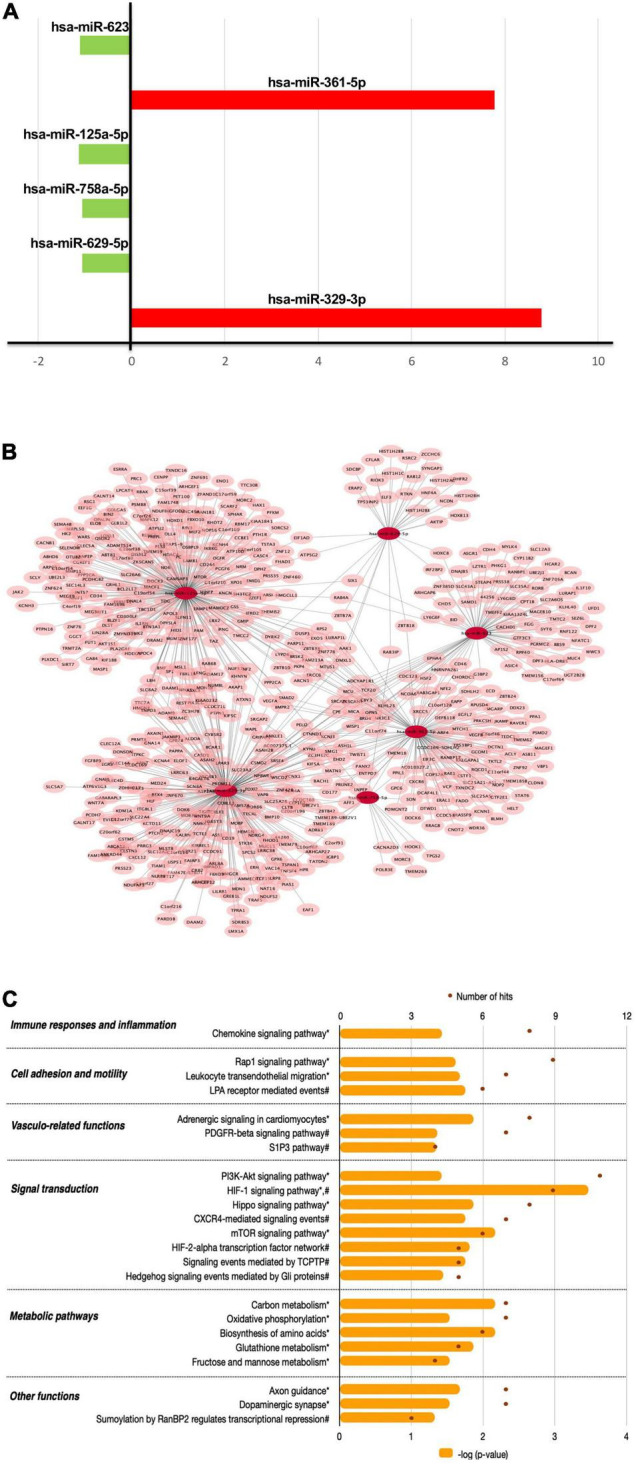
The effects of flavanones in GFJ on miRNA expression in PBMCs with a functional analysis of differentially modulated miRNAs. **(A)** Fold-changes for the identified differentially expressed miRNAs following the consumption of GFJ; **(B)** Network presentation of miRNAs and potential target genes; **(C)** Functional pathway analysis of miRNAs target genes. The orange histogram bars depict *p*-values of the identified significant pathways, and brown dots the number of genes in each pathway. Histogram data obtained using BioCarta (†), Kyoto Encyclopedia of Genes and Genomes (*) and NCI Pathways (#) databases through GeneTrail2.

The miRNA expression analysis showed that chronic consumption of GFJ could differentially modulate the expression of 6 miRNAs in PBMCs compared to the matched control drink without flavanones. Of these miRNAs, two were identified as upregulated (hsa-miR-623, hsa-miR-629-5p) and four as downregulated (hsa-miR-361-5p, hsa-miR-125a-5p, hsa-miR-758-5p, hsa-miR-329-3p), with the fold change varying from −1.13 to 8.78 (Figure 6A).

The obtained differentially expressed miRNAs were further analyzed to identify their potential target genes and possible biological effects associated with their modulation. The MIENTURNET tool analysis revealed 591 gene targets (experimentally validated and computationally predicted) of the identified differentially expressed miRNAs (Figure 6B). Further bioinformatics analyses using GeneTrail2 showed that the identified gene targets of differentially expressed miRNAs are involved in regulating different cellular functions. The majority of overrepresented pathways are involved in signal transduction (Figure 6C), including PI3K-Akt signaling pathway, HIF-1 signaling pathway, Hippo signaling pathway and CXCR4-mediated signaling events. Overrepresented pathways were also associated with cell migration, like Rap1 signaling pathway and Leukocyte transendothelial migration, as well as with immune responses and inflammation, represented by Chemokine signaling pathway. Significant pathways also included those related to vascular function, including Adrenergic signaling in cardiomyocytes and PDGFR-beta signaling pathway as well as metabolic pathways, including Carbon metabolism and Oxidative phosphorylation.

### Integration of mRNA and miRNA Omics

**FIGURE 7:**
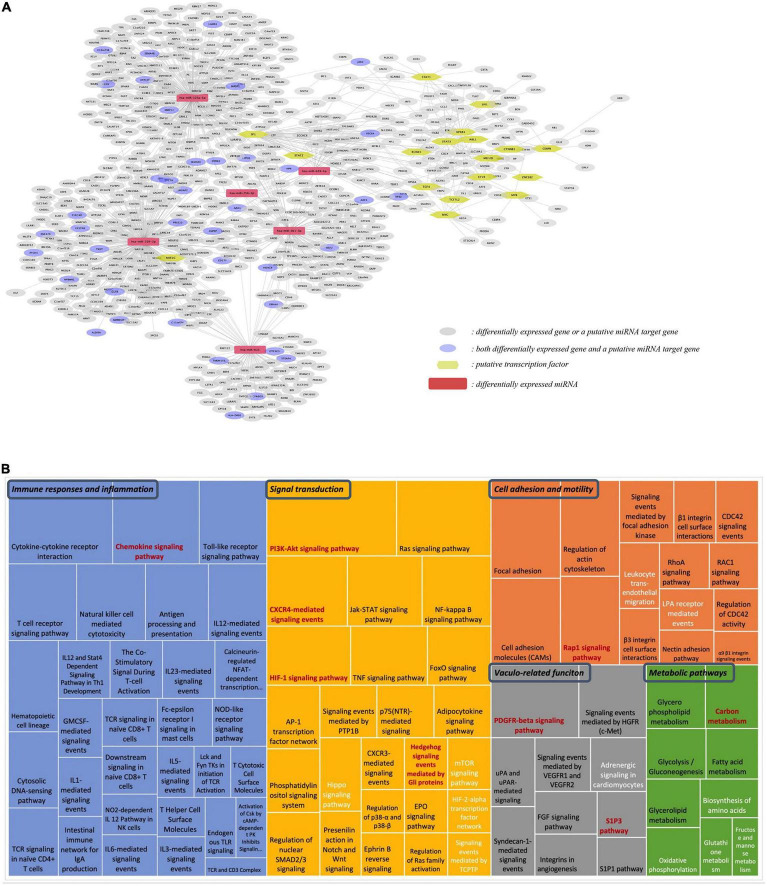
Integration analysis of differentially expressed protein-coding genes and miRNAs. **(A)** Network of the identified putative transcription factors, differentially expressed miRNAs, differentially expressed genes and the identified putative gene targets of differentially expressed miRNAs; **(B)** Comparison of pathways identified from differentially expressed genes and target genes of differentially expressed miRNAs. The size of each rectangle is proportional to the number of genes in the pathway. Black letters: pathways identified from differentially expressed protein-coding genes; white letters: pathways from differentially expressed miRNAs; red letters: pathways in common for both protein-coding and miRNA genes.

Our next step was to integrate different omics data, including differentially expressed protein-coding genes, differentially expressed miRNAs and their targets, and identified transcription factors into one network (Figure 7A). This analysis shows that differentially expressed genes, both protein-coding and miRNAs, form one network of interactions, suggesting an important network of genomic modifications induced by the consumption of flavanones in GFJ in humans.

Comparison of the identified gene targets of miRNA significantly modulated by GFJ flavanones with differentially expressed genes showed 33 genes in common. Comparison of overrepresented pathways associated with miRNA gene targets with those of differentially expressed genes revealed 9 pathways in common (Figure 7B). These pathways were associated with immune response and inflammation (Chemokine signaling pathway), cell adhesion and motility (Rap1 signaling pathway), signal transduction (PI3K-Akt signaling pathway, HIF-1 signaling pathway, CXCR4-mediated signaling events and Hedgehog signaling events mediated by Gli proteins), vasculo-related functions (PDGFR-beta signaling pathway and S1P3 pathway), and metabolism (Carbon metabolism).

### A Subset of Differentially Expressed Genes Can Be Correlated With Changes in Pulse-Wave Velocity

**FIGURE 8:**
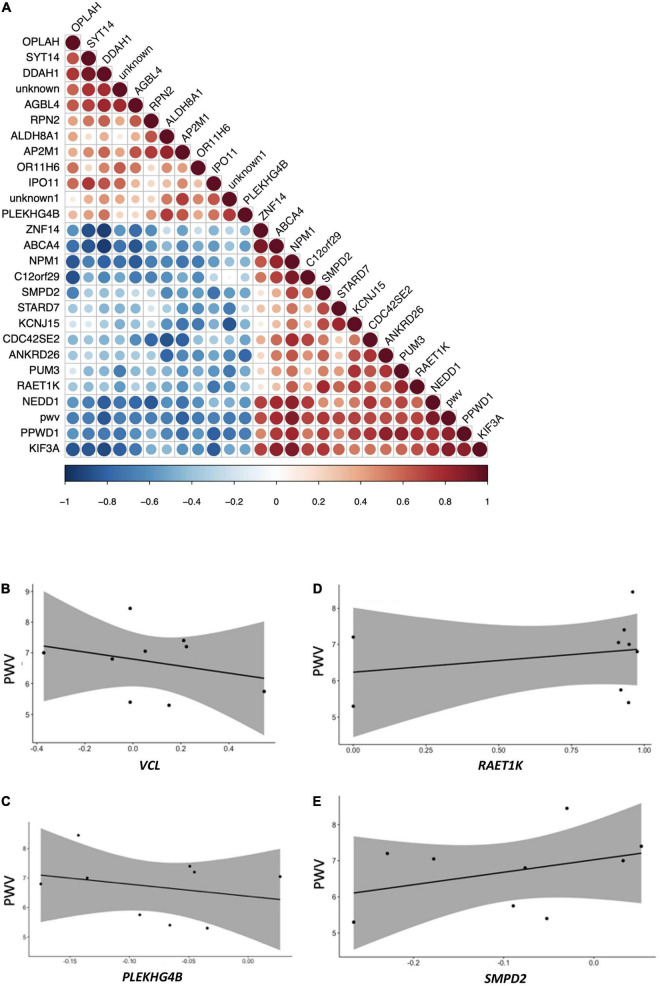
Pearson correlation analysis between modulation in the expression of genes and changes in pulse wave velocity of the same volunteers. **(A)** Correlation plot indicating positive and negative correlations, **(B)** correlation plot between pulse wave velocity and *VCL*; **(C)** correlation plot between pulse wave velocity and *RAET1K*; **(D)** correlation plot between pulse-wave velocity and *PLEKHG4B*; **(E)** correlation plot between pulse wave velocity and *SMPD2*. All *p*-values <0.05.

Among genes identified in this nutrigenomic study as differentially expressed following chronic consumption of GFJ flavanones, 24 genes were identified as significantly correlated with the observed changes in PWV (measured in the original trial) (14) (Figure 8A). Of these genes, the strongest correlations were observed for *NPM1, GLYATL1, PPWD1, DDAH1, PCYT2*, and *ABCA4* (Supplementary Table 3). Among these genes, some showed negative correlation, such as *VCL* (Figure 8B) or *PLEKHG4B* (Figure 8C), and other positive correlations, such as *RAET1K* (Figure 8D) or *SMPD2* (Figure 8E), with observed changes in PWV.

Analysis of pathways associated with the identified significantly correlated genes, using the GeneTrail2 tool, showed that these genes are implicated in pathways like adherent junctions and cell signaling pathways, including VEGFR1 and VEGFR2 and PDGFR-beta signaling pathway.

### Correlation With Gene Expression Changes Associated With Vascular Dysfunction

The next step in the analysis was to examine potential associations between genes identified as having expression modulated by GFJ flavanone consumption and those related to some human diseases. For this purpose, the Comparative Toxicogenomics Database was used. This analysis showed that among the most significant diseases associated with GFJ-modulated genes are CVD (*p*-value <10^–29^), vascular diseases (p-value <10^–25^), heart diseases (*p*-value <10^–18^) or cerebrovascular disorders (*p*-value <10^–7^). Comparison of the identified differentially expressed genes in the present study with genes associated with CVD development (identified from the DisGeNET database) showed over 220 genes in common with cardiovascular, heart, and cerebrovascular diseases. These analyses suggest that genomic modifications induced by GFJ flavanone are related to these diseases, nonetheless we cannot identify if changes observed will result in development or prevention of these disorders.

**FIGURE 9:**
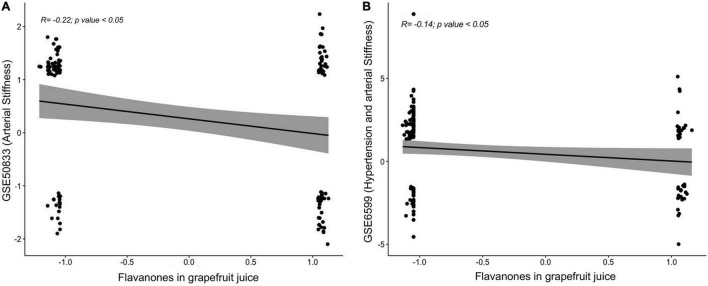
Correlations between global gene expression profile from volunteers who consumed grapefruit juice and gene expression profile associated with an increase in **(A)** arterial stiffness and **(B)** hypertension and arterial stiffness obtained from Gene Expression Omnibus (GEO) repository.

To identify whether the GFJ-induced modulations in gene expression are associated with disease prevention, correlation analyses were performed between the identified modulated genes and genes associated with CVD. Gene expression data were obtained from GEO datasets^13^ and differentially expressed mRNAs were correlated with those modulated by GFJ consumption using the Pearson correlation. This analysis showed that the gene expression profile associated with an increase in arterial stiffness with aging (GSE50883) is inversely correlated with the gene expression profile after consumption of GFJ in our study (Figure 9A). Similarly, a negative correlation between gene expression profile in old monkeys presenting hypertension and arterial stiffness (GSE6599) and genes identified as modulated by regular GFJ consumption was also observed (Figure 9B). These analyses suggest that GFJ flavanones modulate the expression of genes in the opposite way to the expression of genes associated with vascular dysfunctions, suggesting that flavanone-induced gene expression profile is protective regarding cardiovascular health.

## Discussion

**FIGURE 10:**
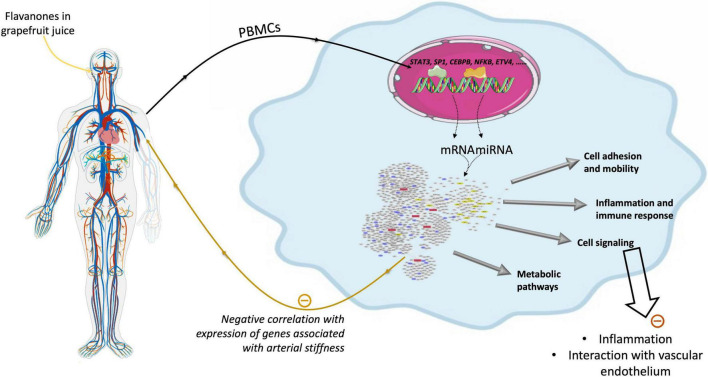
Schematic presentation of genomic modifications induced by consumption of flavanones in grapefruit juice and its impact on vascular health.

As summarized in Figure 10, this human study based on microarray analysis showed that chronic consumption of GFJ naturally rich in flavanones for 6 months induced changes in the expression of protein-coding genes and miRNAs in postmenopausal women’s PBMCs. The observed differentially expressed genes by GFJ flavanones are involved in biological processes that include inflammation, immune responses, cell motility, vascular-related functions and signal transduction and are important for vascular homeostasis. The obtained results also agree with the capacity of grapefruit flavanones to protect from arterial stiffness, as we have reported previously in the same study population (14).

Previous human nutrigenomic study focusing on orange flavanones in healthy overweight men demonstrated that the 4-week consumption of orange juice naturally rich in hesperidin modulated the gene expression in leukocytes with a shift toward a more anti-inflammatory and antiatherogenic gene expression profile (26). In animal studies, grapefruit extract exhibited anti-obesogenic and antihyperglycemic effects by modulating the expression of genes implicated in lipid metabolism and inflammation, respectively (27, 28). Moreover, the capacity of grapefruit flavanones to alter the expression of different genes related to inflammatory responses, cell mobility and lipid metabolism have been reported in animal models of atherosclerosis, obesity and type-2 diabetes, as well as *in vitro* studies (15, 29–31).

In the present work, the bioinformatics analyses of genes observed as differentially modulated by chronic consumption of GFJ flavanones identified a group of gene networks and pathways involved in controlling inflammatory responses, cell adhesion and motility that are major underlying mechanisms of cardiometabolic disorders. For example, we detected the modulation of genes encoding TNFα receptors that mediate proinflammatory responses and contribute to immunomodulation. Previously, increased circulating TNFα receptor levels were associated with higher risks of cardiovascular events and mortality in subjects with stable coronary heart disease (32). Moreover, *TNFRSF1A* and *TNFRSF10B* PBMCs expression and plasma levels have been positively correlated with PWV in CAD patients (33, 34). Thus, the observed downregulation of these receptors in our study could be linked with the potential anti-inflammatory properties of GFJ and previously reported improvements in arterial stiffness in these subjects (14).

Several of the observed differently modulated genes by GFJ flavanones are coding for different chemokines and their receptors that are involved in chemotaxis. These molecules regulate inflammation and the formation of atherosclerotic lesions in the arterial wall by promoting immune cell activation and migration. Also, through their role in inflammation and vascular dysfunction, chemokines are implicated in the etiology of hypertension (35). We observed reduced expression of *CXCL10* and *CXCL1* involved in the trafficking of monocytes/T cells and leukocytes, respectively. These chemokines are upregulated in human atheromas and plasma of hypertensive or CAD patients, while their inhibition reduces atherosclerotic lesion formation and immune cell accumulation in animal models (36). The observed downregulation of these genes in our study corroborated their previously reported downregulation in activated macrophages after the treatment with naringenin (37). We also observed the downregulation of genes encoding monocyte chemoattractant protein-1 (MCP-1 or CCL2) and its receptor CCR2 that are involved in monocyte/macrophage migration and are found upregulated in atherosclerosis (35). Moreover, increased plasma MCP-1 levels were reported in subjects with young-onset hypertension and positively correlated with PWV (38). In animal models of atherosclerosis (39) and hypertension (40), the genetic deletion of MCP-1 or its receptor resulted in reduced inflammation and monocyte/macrophage infiltration in the aorta. These effects were also observed and accompanied by downregulation of the aortic *CCL2* expression after dietary supplementation with naringin (41). Thus, these data suggest that the modulation of the expression of chemokines and their receptors could be one of the mechanisms underlying the cardioprotective action of GFJ flavanones.

The consumption of flavanones in GFJ also modulated the expression of genes encoding cell adhesion molecules that mediate the interactions between immune cells and vascular endothelium and contribute to the initial stages of atherosclerosis. For example, we observed the downregulation of a gene encoding platelet endothelial cell adhesion molecule 1 (PECAM-1) that promotes monocyte transmigration through endothelial cells. Blocking monocyte’s PECAM-1 with antibodies was shown to reduce transendothelial migration of these cells (42). Moreover, genetic deletion of *PECAM1* in apolipoprotein E deficient (apoE^–/–^) mice showed a significant decrease in atherosclerosis development compared to apoE^–/–^ controls (43). We also observed downregulation of gene encoding integrin subunit beta 2 (ITGB2), integrin family member expressed on immune cells that mediates their adhesion and transendothelial migration. Genetic deletion of this gene was reported to reduce aortic lesion size in mice (44), while mutations in *ITGB2* cause leukocyte adhesion deficiency (43). Furthermore, integrins can also affect vascular function by acting on angiogenesis (45), which is promoted in atherosclerosis. GFJ consumption also downregulated the expression of a gene encoding vascular endothelial growth factor (VEGF) A that plays an important role in endothelial function and angiogenesis. VEGFA expressed by macrophages and T-lymphocytes was shown to stimulate endothelial cells to produce chemokines, attracting monocytes and promoting their transmigration by elevating the endothelial layer’s permeability (46). Finally, we also observed that GFJ affected the expression of genes coding for Ras and Rho family of small GTPases and associated proteins (e.g., *RAPH1, RHOB*, *RHOD*, *ARHGEF40*, *ARHGEF42*) that are involved in cytoskeletal reorganization, contractility and cell motility (47, 48). Therefore, the observed expression profiles of the above-presented genes by GFJ flavanones suggest reduced immune cell trafficking and vasculoprotective effects.

Among the identified modulated genes were also those involved in several signaling pathways, particularly PI3K-Akt signaling (with 26 genes), which manages survival, proliferation, and migration of monocytes and impacts atherosclerosis development. Aberrant PI3K-Akt signaling has been described in CVD and related comorbidities, and its targeting suggested as a potential tactic in the prevention and treatment of atherosclerosis (49). Accordingly, dietary naringin modulated the expression of several genes involved in PI3K-Akt signaling pathway and reduced aortic plaque formation (15) in an animal model of atherosclerosis, suggesting this signaling as one of the targets of this grapefruit flavanone’s cardioprotective effects.

Our bioinformatic analysis of differentially expressed genes allowed us to identify several potential transcription factors, whose activities could be modulated by flavanone consumption and could be involved in the observed genomic modifications. Moreover, our *in silico* docking analysis also suggested that flavanone metabolites present a high potential to bind to STAT3, but also STAT1. It has been described that risk factors involved in atherosclerosis and plaque development can trigger activation of these transcription factors, which can promote inflammation, especially in immune and vascular cells during atherosclerosis (50). Moreover, *in vivo* studies demonstrated increased STAT3 activation in monocytes of hypertensive subjects as well as increased aortic and renal infiltration of monocytes and derived cells, with activated STAT3 in mice with experimental hypertension (51). Our *in silico* analysis also suggested that naringin metabolites present a high binding capacity to JAK2 involved in JAK2/STAT signaling pathway. It has been observed that the JAK2/STAT3 pathway plays an important role in regulating age- and hypertension-associated rise of aortic stiffness (52). *In silico* analysis also suggested that naringin metabolites could interact with Akt as well as ERK1 cell signaling proteins, both of which have been suggested to play a role in vascular function (53, 54). Further experimental studies are needed to assess if these potential binding would result in activation or inhibition of the target proteins. Taken together, these bioinformatic analyses revealed important transcription factors potentially involved in the genomic modulation by naringin-rich juice consumption in humans and may present key regulatory proteins/genes underlying their health properties.

In the present study, we also demonstrated that chronic consumption of GFJ flavanones modulated the expression of 6 miRNAs in PBMCs, revealing for the first time the capacity of these compounds to act on these post-transcriptional regulators in humans. Citrus flavanones have been reported previously to modulate miRNA expression in preclinical models (20, 55, 56), however, data on the impact of these citrus bioactives on miRNA are still limited. MiRNAs have been identified as the important regulators of vascular function, which deregulation seems to contribute to vascular dysfunction and CVD development (57). Among miRNAs identified in this study as downregulated by GFJ were miR-758-5p and miR-361-5p, previously found upregulated in atherosclerosis (58, 59). miR-361-5p was found upregulated in acute coronary syndrome patients’ sera, positively correlated with the levels of markers of endothelial dysfunction and represented an independent risk factor for the occurrence of major adverse cardiac events (60). We also observed reduced miR-125a-5p expression, which higher circulating levels were previously linked with hyperlipidemia, hyperglycemia and acute ischemic stroke (61, 62). Another downregulated miRNA was miR-329-3p, whose levels were increased in PBMCs of CAD patients and positively correlated with the level of arterial stenosis (63). Downregulation of this miRNA was shown to promote vascular endothelial cell proliferation and blood flow recovery after ischemia (64) as well as control carotid atherosclerotic plaque development (65). After GFJ naringin consumption, we also observed upregulation of miR-623 and miR-629-5p. Although mainly described in cancer, these miRNAs are implicated in the regulation of cell motility (66) and could play a role in vascular function. Our bioinformatic analysis also showed that target genes of the identified differentially expressed miRNAs are involved in controlling inflammation, immune response and interaction with vascular endothelium, processes regulating vascular function. Taken together, the expression profiles of miRNA modulated by GFJ naringin suggest vasculoprotective effects and potential protection against CVD development.

We have shown previously in the same volunteers that the consumption of GFJ for 6 months significantly reduced PWV (14). PWV is widely used to diagnose aortic stiffness, which is regarded as an important cardiovascular risk factor contributing to vascular dysfunction. Here, we performed correlation analysis between changes in the expression of genes and changes in PWV of the same volunteers. Among the genes identified as negatively correlated with PWV is *DDAH1* (Dimethylarginine Dimethylaminohydrolase 1). This gene codes for an enzyme that regulates nitric oxide generation by controlling cellular concentrations of methylarginines, which in turn inhibit nitric oxide synthase activity. Interestingly it has been shown that the person having mutation in this gene, inactivating protein activity, had a significantly elevated risk for CVD and a tendency to develop hypertension (67). Moreover, the GWAS association study observed a significant association (1 × 10^–9^) between polymorphism in this gene (rs12034319-A) and pulse pressure measurement, a difference between systolic blood pressure and diastolic blood pressure, which is one of the early risk factors of CVD [GCST006629; (68)].^14^ Correlation analysis also revealed negative correlation of *ANKRD26* (Ankyrin Repeat Domain 26) with PWV. A GWAS analysis on over 12000 volunteers showed a significant association (5 × 10^–6^) between a mutation in this gene (rs7081476) and CAD, diastolic blood pressure, systolic blood pressure, heart failure and mortality (69).

Interrogation of genomic databases also suggested that modulation of the expression of genes by flavanones in GFJ, taking into account their expression profile, suggests that these bioactives could play a role in the prevention of the development of diseases, particularly CVD, together with other bioactives, diets or exercise. Correlation analysis between the observed gene expression profile of flavanones with gene expression profile in individuals presenting arterial stiffness suggested a protective effect regarding this risk factor due to observed opposite expression profiles. This observation agrees with previous studies demonstrating the cardiovascular health effects of flavanones (17, 70). This analysis also suggests that global gene expression profiling in PBMCs, the most easily accessible cells in clinical trials, could present an interesting method to predict the health properties of bioactives, nutrients or diets.

## Conclusion

This study showed for the first time that flavanone consumption in GFJ could induce changes in the gene expression profile of protein-coding genes but also of protein non-coding genes, microRNAs. These genes form a complex network of interactions that regulate cellular processes, particularly those involved in inflammation, cell adhesion and cell mobility. Expression of some of the genes has been observed as correlated with changes in PWV detected in the same volunteers. Moreover, global change in expression of genes is negatively correlated with gene expression profile observed in patients with arterial stiffness and hypertension. Therefore, the results of this study suggest that regular consumption of flavanone in grapefruit juice could modulate the expression of genes that could contribute to the prevention of vascular dysfunction and, consequently, the development of CVD.

## Data Availability Statement

Data are publicly available at GEO database: Series record GSE202084, https://www.ncbi.nlm.nih.gov/geo/query/acc.cgi?acc=GSE202084.

## Ethics Statement

The studies involving human participants were reviewed and approved by Local Human Ethics Committee (CPP Sud-Est VI, France). The patients/participants provided their written informed consent to participate in this study.

## Author Contributions

DM, CD, and CM contributed to the conception and study design. IK, KC-J, NB-C, and DM carried out the research work, prepared the draft manuscript, performed the analysis and interpretation of results. All authors approved the final version of the manuscript.

## Conflict of Interest

The authors declare that the research was conducted in the absence of any commercial or financial relationships that could be construed as a potential conflict of interest.

## Publisher’s Note

All claims expressed in this article are solely those of the authors and do not necessarily represent those of their affiliated organizations, or those of the publisher, the editors and the reviewers. Any product that may be evaluated in this article, or claim that may be made by its manufacturer, is not guaranteed or endorsed by the publisher.

## Footnotes

1
https://www.ensembl.org/index.html

2
https://www.metaboanalyst.ca/

3
http://bioinformatics.sdstate.edu/go/

4
https://portal.genego.com

5
https://genetrail2.bioinf.uni-sb.de/

6
https://string-db.org/

7
https://maayanlab.cloud/Enrichr/

8
https://mcule.com/apps/1-click-docking/

9
https://pubchem.ncbi.nlm.nih.gov

10
http://userver.bio.uniroma1.it/apps/mienturnet/

11
https://cytoscape.org/

12
https://www.r-project.org/

13
https://www.ncbi.nlm.nih.gov/geo/

14
https://www.ebi.ac.uk/gwas/home
