# Supplementary material for: Grapefruit Juice Flavanones Modulate the Expression of Genes Regulating Inflammation, Cell Interactions and Vascular Function in Peripheral Blood Mononuclear Cells of Postmenopausal Women
Source: Front Nutr. 2022 May 26;9:907595. doi: 10.3389/fnut.2022.907595 (PMC9178201; doi:10.3389/fnut.2022.907595)

Supplementary figure S1

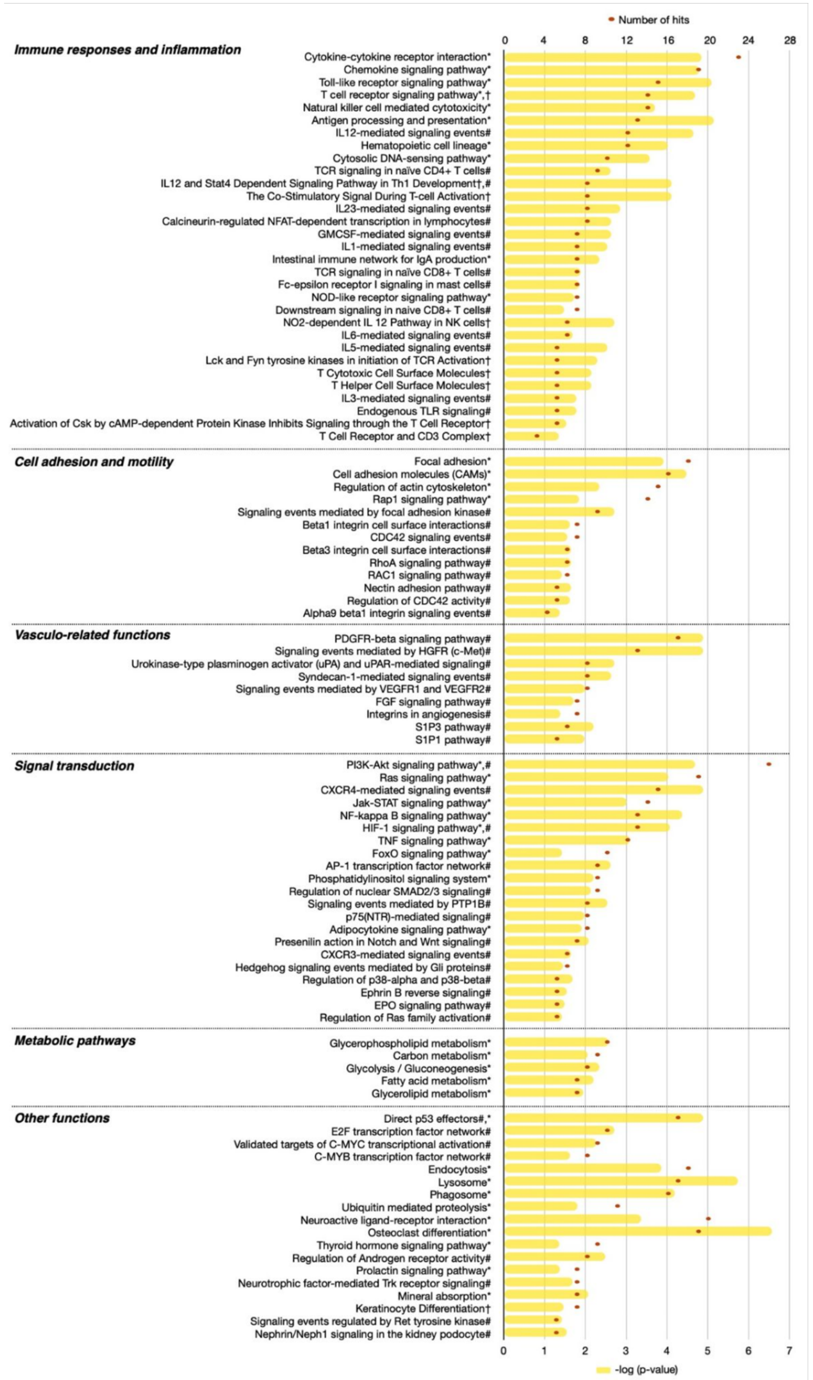

Supplementary figure S2A

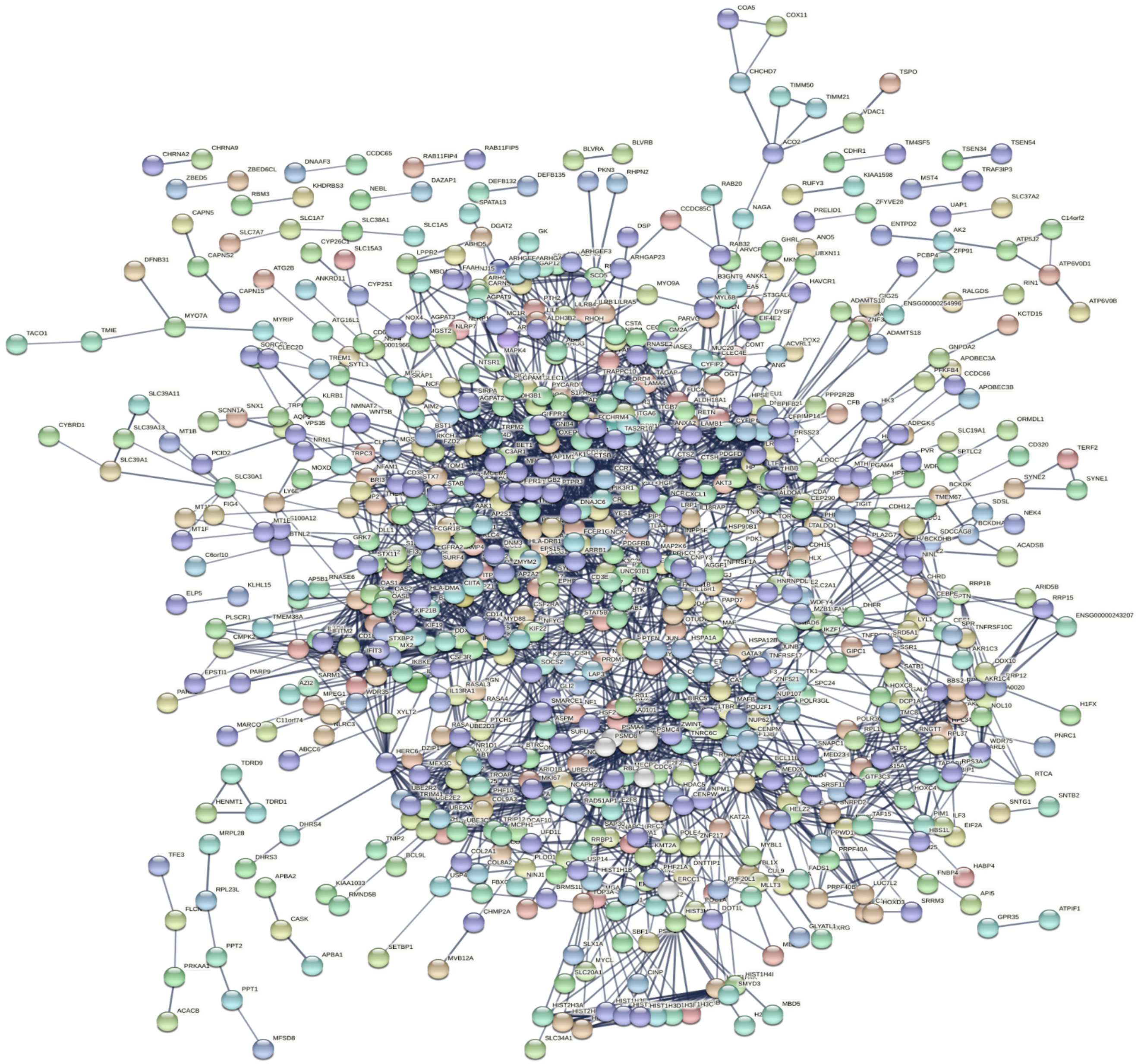

Supplementary Figure S2B

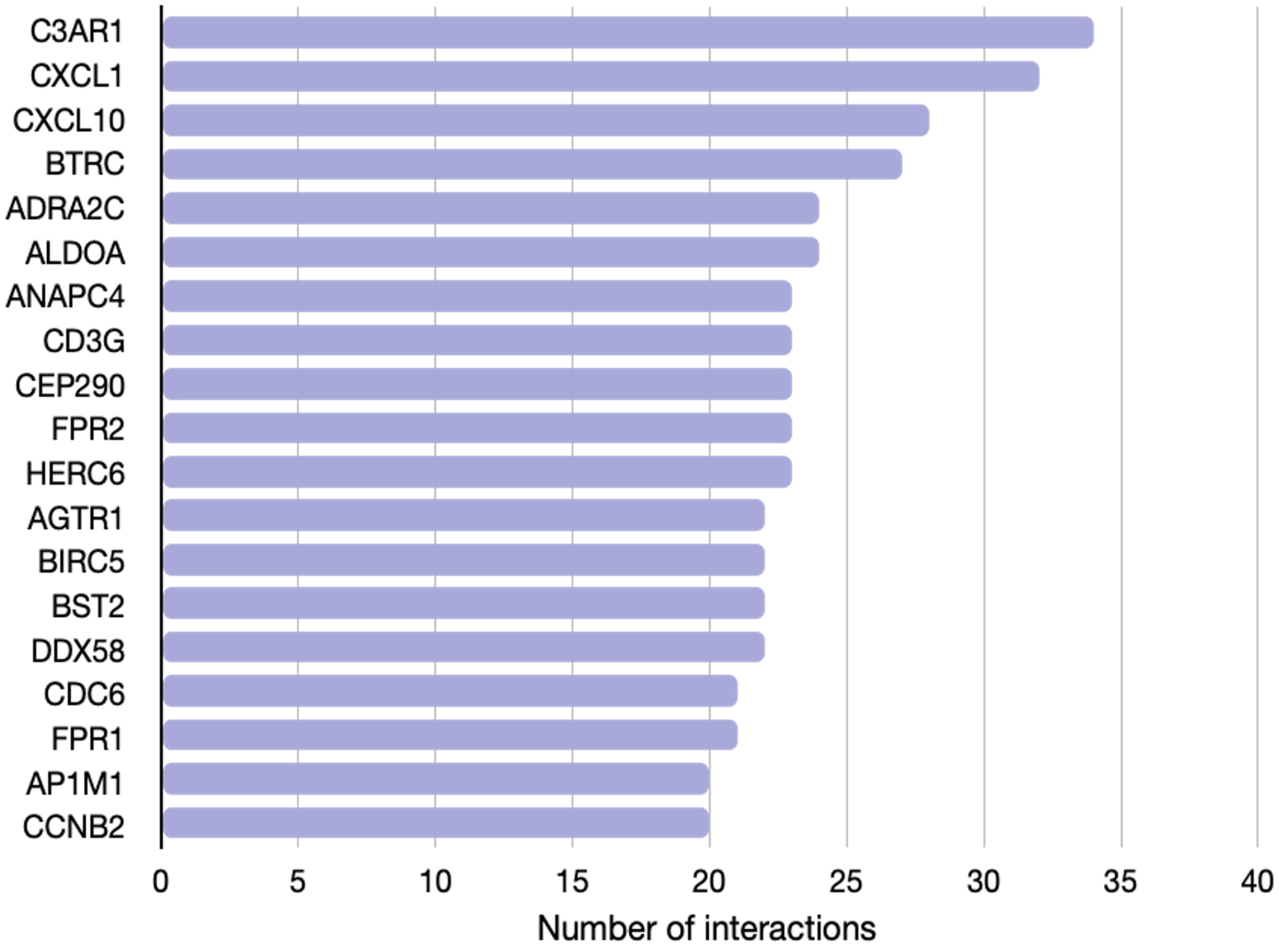

Supplementary Figure S3

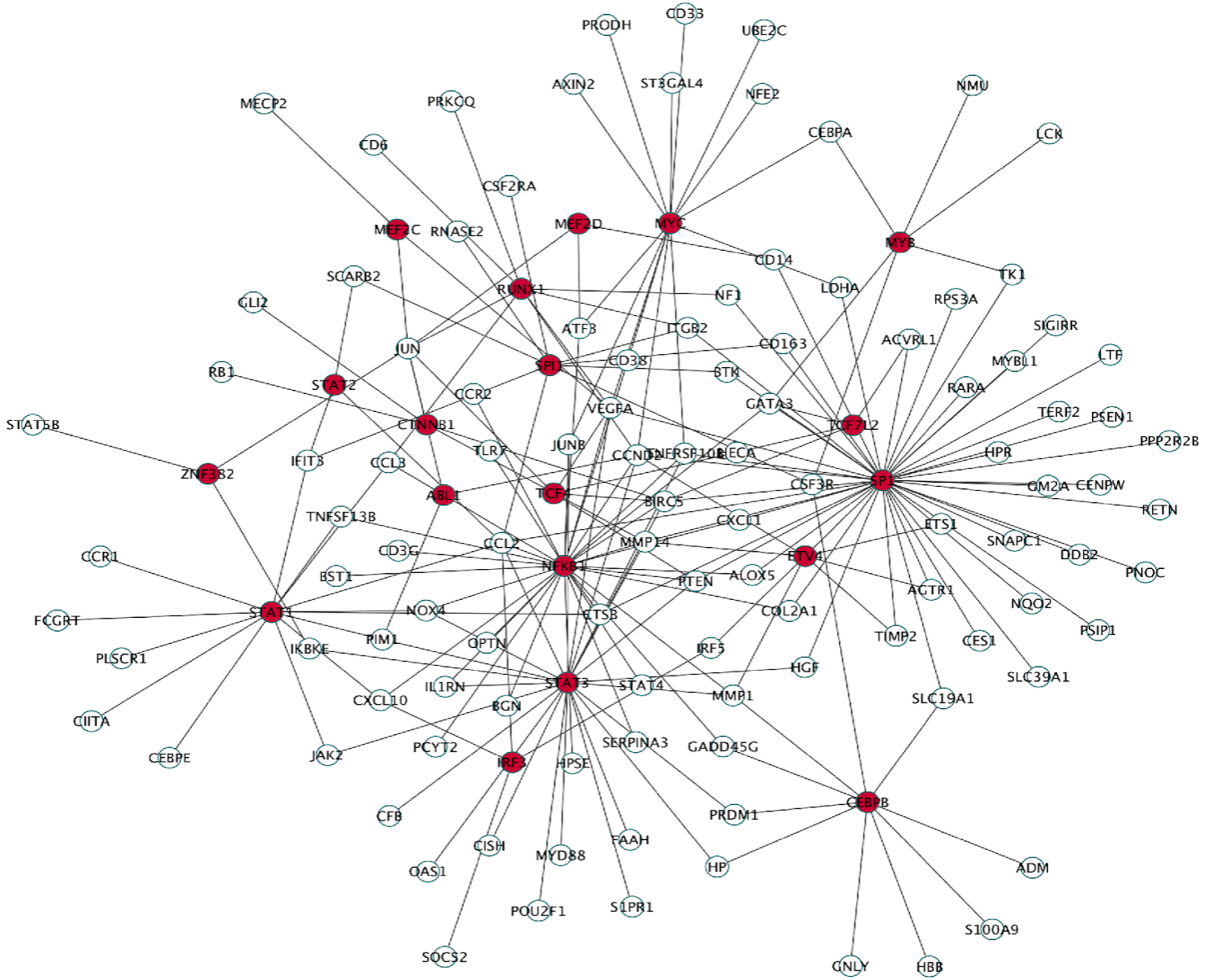

Supplement: Supplementary file 1 [file Data_Sheet_1.PDF]
